# [^18^F]FEPPA a TSPO Radioligand: Optimized Radiosynthesis and Evaluation as a PET Radiotracer for Brain Inflammation in a Peripheral LPS-Injected Mouse Model

**DOI:** 10.3390/molecules23061375

**Published:** 2018-06-07

**Authors:** Nicolas Vignal, Salvatore Cisternino, Nathalie Rizzo-Padoin, Carine San, Fortune Hontonnou, Thibaut Gelé, Xavier Declèves, Laure Sarda-Mantel, Benoît Hosten

**Affiliations:** 1 Assistance Publique—Hôpitaux de Paris, Hôpital Saint-Louis, Unité Claude Kellershohn, 75010 Paris, France; nicolas.vignal@aphp.fr (N.V.); nathalie.rizzo@aphp.fr (N.R.-P.); carine.san@aphp.fr (C.S.); thibaut.gele@aphp.fr (T.G.); laure.sarda-mantel@aphp.fr (L.S.-M.); 2 Inserm UMR-S 1144, Faculté de Pharmacie de Paris, Université Paris Descartes, 75006 Paris, France; salvatore.cisternino@aphp.fr (S.C.); xavier.decleves@aphp.fr (X.D.); 3 Assistance Publique—Hôpitaux de Paris, Hôpital Universitaire Necker—Enfants Malades, 75015 Paris, France; 4 Institut Universitaire d’Hématologie, Université Paris Diderot, 75013 Paris, France; fortune.hontonnou@univ-paris-diderot.fr; 5 Assistance Publique—Hôpitaux de Paris, Hôpital Cochin, 75014 Paris, France; 6 Assistance Publique—Hôpitaux de Paris, Hôpital Lariboisière, Médecine Nucléaire, 75010 Paris, France; 7 Inserm UMR-S 942, Université Paris Diderot, 75013 Paris, France

**Keywords:** [^18^F]FEPPA, TSPO, brain inflammation, small-animal PET imaging, radiolabeling, radiotracer metabolism

## Abstract

[^18^F]FEPPA is a specific ligand for the translocator protein of 18 kDa (TSPO) used as a positron emission tomography (PET) biomarker for glial activation and neuroinflammation. [^18^F]FEPPA radiosynthesis was optimized to assess in a mouse model the cerebral inflammation induced by an intraperitoneal injection of *Salmonella enterica* serovar *Typhimurium* lipopolysaccharides (LPS; 5 mg/kg) 24 h before PET imaging. [^18^F]FEPPA was synthesized by nucleophilic substitution (90 °C, 10 min) with tosylated precursor, followed by improved semi-preparative HPLC purification (retention time 14 min). [^18^F]FEPPA radiosynthesis were carried out in 55 min (from EOB). The non-decay corrected radiochemical yield were 34 ± 2% (*n* = 17), and the radiochemical purity greater than 99%, with a molar activity of 198 ± 125 GBq/µmol at the end of synthesis. Western blot analysis demonstrated a 2.2-fold increase in TSPO brain expression in the LPS treated mice compared to controls. This was consistent with the significant increase of [^18^F]FEPPA brain total volume of distribution (*V_T_*) estimated with pharmacokinetic modelling. In conclusion, [^18^F]FEPPA radiosynthesis was implemented with high yields. The new purification/formulation with only class 3 solvents is more suitable for in vivo studies.

## 1. Introduction

The translocator protein of 18 kDa (TSPO) is the most advance target for the non-invasive and translational study of inflammatory processes using positron emission tomography (PET) imaging [1]. TSPO is an outer mitochondrial membrane protein found in many cell types but there is growing literature documenting its restricted brain expression in microglia and astrocytes [2]. Cerebral inflammation is supported mainly by microglial cells, which are the resident immune cells of the central nervous system (CNS) [3]. Microglia are activated in response to endogenous signals such as beta-amyloid plaques, Tau protein [4,5,6], α-synuclein [6,7,8], or exogenous “danger” signals such as traumatic CNS injury [9] or bacterial lipopolysaccharide (LPS) [10]. This cellular activation leads to numerous functional and biochemical changes that involve cell morphology, metabolism, cytokine production and some de novo protein synthesis, for instance an increase in the ionized calcium binding adaptor molecule 1 (Iba1) and TSPO, both used as biomarkers for microglial activation. Microglia can adopt different morphologies and multiple functions: pro-inflammatory or reparative [11,12]. Other glial cells such as astrocytes are activated and may be involved in CNS inflammation [13,14].

Clinical PET imaging for brain inflammation in addition to conventional biological examinations may be a useful tool to support or reject the hypothesis of localized inflammation in the brain, to follow this process during the disease, and to study possible links between systemic and CNS inflammation. Neuroinflammation is known as a major factor in the pathophysiology of many neurodegenerative diseases [15,16,17,18]. TSPO PET studies have been conducted and showed an increase in TSPO signal in patients with Alzheimer disease [19,20], Parkinson disease [21,22], and multiple sclerosis [23,24]. More recently, clinical studies using [^18^F]FEPPA PET have demonstrated a brain inflammation component in some psychiatric diseases such as schizophrenia [25] and major depressions [26]. This technology is currently being applied in humans in psychosis [27,28] or Alzheimer disease [29].

PET imaging of neuroinflammation using TSPO radioligands is rather well documented, particularly for [^11^C]PK11195 and [^18^F]DPA-714. However, ^11^C-labeled radiotracers are more difficult to manage clinically, and [^11^C]PK11195 is characterized by rather broad non-specific binding, which may limit the quantification of the PET signal [30]. Hence specific radiotracers need to be developed and optimized for clinical use. [^18^F]FEPPA is one of the newest TSPO PET radiotracer with greater affinity for its target [31] and one of the two TSPO radioligand being the most advanced in clinical development with [^18^F]DPA-714 for PET imaging of neuroinflammation. Both radiotracers encountered limits to their applications, since it was shown in human three patterns of binding affinity (based on genetic polymorphism) [32] as well as an absence of reference tissue for their quantification. The radiosynthesis of [^18^F]FEPPA was first described by Wilson et al. in 2008 [31]. In this report, [^18^F]FEPPA purification was assessed using semi-preparative HPLC with methanol and formic acid followed by drying and recovery with ethanol and water, which could introduce traces of toxic solvents into the finished product and add steps to the radiotracer formulation process. In 2013, the same team modified their [^18^F]FEPPA radiosynthesis protocol by replacing the last step of the formulation with a cartridge capture followed by rinsing and ethanol/water elution [33]. An other team has used acetonitrile/ammonium formiate for HPLC purification which needs formulation with cartridge [34]. For human use, pharmaceutical products must limit the use of class 2 solvents (International Council for Harmonisation of Technical Requirements for Pharmaceuticals for Human Use Q3C) such as methanol or acetonitrile. To achieve this, Huang et al. [35] describe a radiosynthesis with an HPLC purification with only ethanol and water but with a great decrease in radiochemical yield.

PET studies of neuroinflammation have mostly investigated pathologies with an initially central inflammatory process, while very few have investigated those induced by a peripheral inflammatory stimulus. To our knowledge, the use of [^18^F]FEPPA to monitor brain inflammation induced by intraperitoneal (ip) LPS injection, has never been reported. Peripheral LPS injection is often used to induce neuroinflammation in mice [10]. However, the neuroinflammatory response may differ depending on the mouse and/or LPS strains [36]. Although the effects of *Salmonella enterica serovar typhimurium* LPS have been suggested to be more severe than *E. coli* LPS [37], LPS-injection models have mainly used *E. coli* LPS [10,36].

The objective of this work was to develop and optimize the fully automatized radiosynthesis of [^18^F]FEPPA on the Allinone automat (Trasis^®^) with solvents more suitable for human use (class 3, ICH Q3C). The aim of this study was also to quantify metabolism of [^18^F]FEPPA in mice which is currently unknown, and to evaluate this radiotracer in a mouse model of neuroinflammation induced by peripheral *Salmonella enterica serovar typhimurium* LPS ip injection [38].

## 2. Results

### 2.1. Automated Radiosynthesis and Characterization of [^18^F]FEPPA

**Figure 1:**
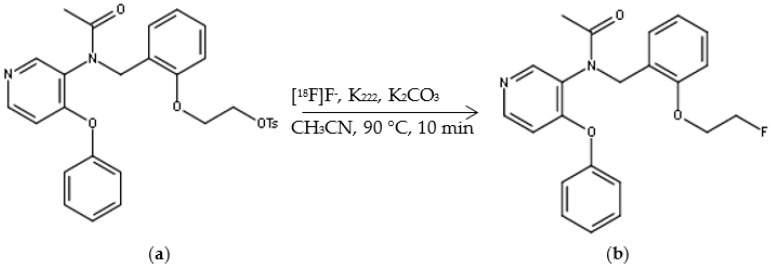
(**a**) FEPPA Precursor (2-(2-((*N*-4-phenoxypyridin-3-yl)acetamido)methyl)phenoxy)ethyl 4-methylbenzenesulfonate); (**b**) [^18^F]FEPPA.

[^18^F]FEPPA was synthetized according to previously published methods [31,33] with some modifications and by using the AllInOne^®^ module (Figure 1). [^18^F]FEPPA was produced with a non-decay corrected radiochemical yield of 34% ± 2% within 55 min (*n* = 17) from end of bombardment. 

**Figure 2:**
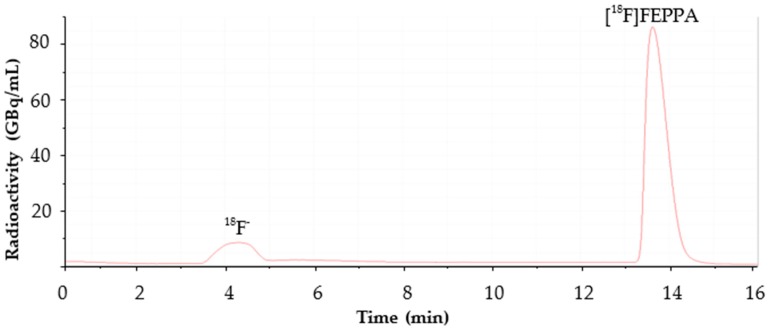
Semi-preparative HPLC radiochromatogram.

The semi-preparative HPLC mobile phase was optimized from methanol and formic acid to ethanol and phosphoric acid. This new HPLC conditions allowed us to bypass the reformulation step and decrease the retention time (Figure 2) from 26 min [33] to 14 min, with a satisfactory separation of the [^18^F]FEPPA from its impurities. The useful fraction of the semi-preparative HPLC was collected in a vial containing 1.5 mL of sodium bicarbonate 8.4%. This allowed the final product to reach a physiological pH (between 6.8 and 7.3), which is more suitable for injection and stability.

**Figure 3:**
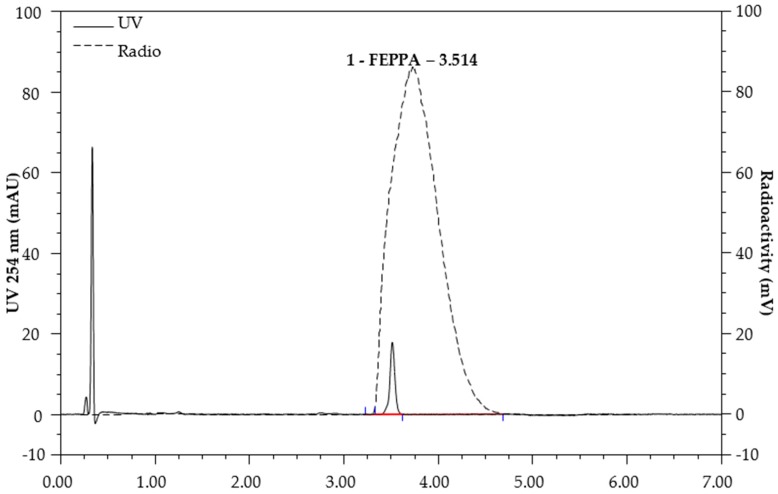
Analytical HPLC chromatogram (radio and UV).

Quality control of the final product confirms that our semi-preparative HPLC allows a complete separation of [^18^F]FEPPA from synthesis impurities (Figure 3). Retenrion time for FEPPA and the precursor are respectively 3.5 and 4.6 min (resolution factor of 10.2). Our quality control method needs less amount of the final product (5 µL instead of 20 µL with a HPLC column similar to the method by Vasdev et al. [33]) and therefore reduces operator exposition during the procedure. Molar activity and activity concentration of the final product ranged respectively from 74 to 410 GBq/µmol and from 2.3 to 4.8 GBq/mL at the end of synthesis. For all produced batches, chemical and radiochemical purity were greater than 99% and the latter was maintained >98% over a period of 6 h in saline. 

### 2.2. Biodistribution

**Figure 4:**
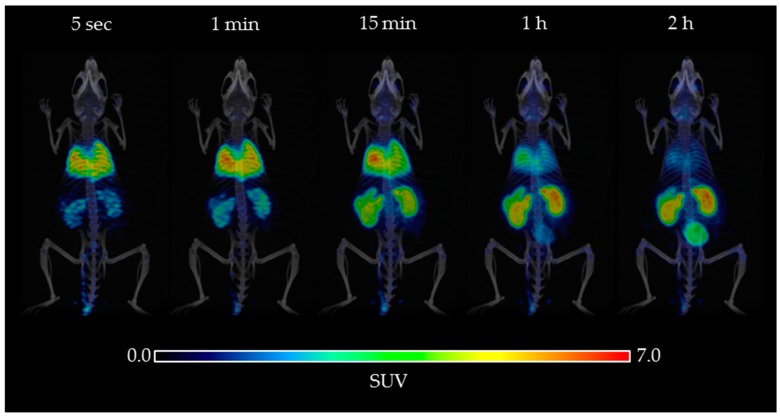
[^18^F]FEPPA 3D PET/CT imaging from tracer injection to 120 min after in a C57BL/6 mouse. Image at 5 s, 1 min, 15 min, 1 h ,and 2 h post-injection.

Figure 4 illustrates the whole body distribution of [^18^F]FEPPA over time from radiotracer injection up to 120 min, using microPET imaging coupled with computerized tomography (CT). Visual analysis demonstrated physiologic [^18^F]FEPPA rapid uptake in heart, lung, spleen, and kidney excretion. In contrast, brain uptake of [^18^F]FEPPA is low. At 15 min mean SUV were: 3.7 for lung, 5.1 for heart, 3.8 for kidney and 0.5 for brain. As expected, [^18^F]FEPPA distribution follow the basal TSPO distribution in mice which is found in most tissues and specially in heart and kidney [39] like [^18^F]DPA-714 [40]. The brain TSPO distribution explains the lack of reference tissue and the need for kinetic modelling for quantification.

### 2.3. TSPO Expression in Brain by Western Blot

**Figure 5:**
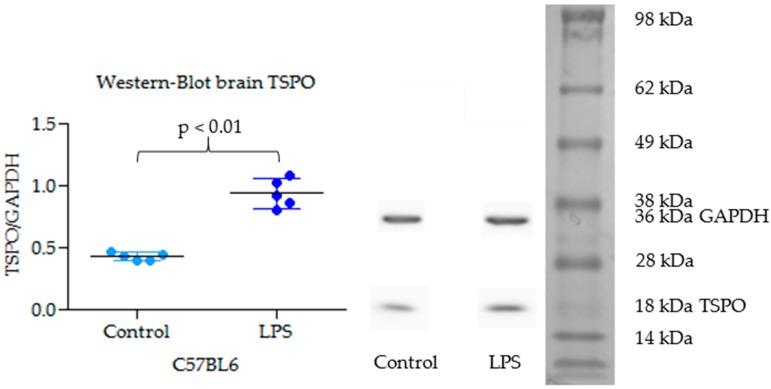
TSPO brain expression in C57Bl6 mice for control and lipopolysaccharides (LPS) conditions.

TSPO expression is 2.16-fold greater in whole brain of LPS injected mice (mean TSPO/GAPDH = 0.93) as compared to control mice (mean TSPO/GAPDH = 0.43) (*p* = 0.004) (Figure 5). TSPO band has been compared to molecular weight scale and confirmed at 18 kDa. GAPDH has been used as loading reference. 

### 2.4. Metabolism Study

**Figure 6:**
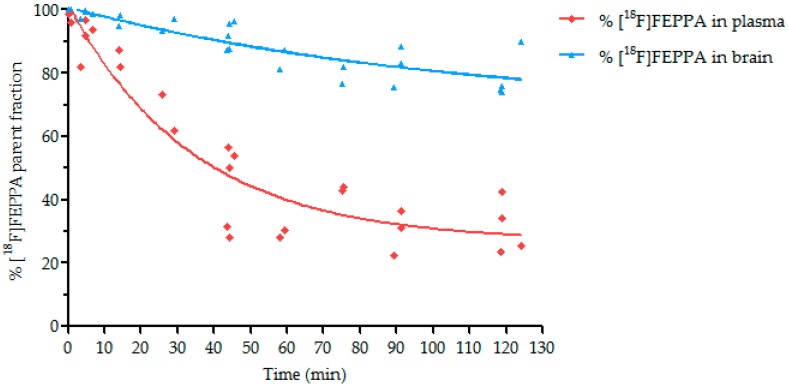
Percentage of parent fraction (unmetabolized) of [^18^F]FEPPA in plasma and brain for control condition.

Metabolism study has shown a rapid decrease of the parent fraction in plasma (60% at 30 min) (Figure 6), with roughly 15% of the metabolite observed 15 min after injection, and 65% of the metabolite 120 min after radiotracer injection. In the brain, a lower metabolism was observed (96% and 77% of the parent fraction, respectively, at 15 and 120 min after [^18^F]FEPPA injection). Only one metabolite was detected other than [^18^F]FEPPA in both plasma and brain.

The percentage of parent fraction determined in brain was used to obtain the time activity curve of unmetabolized [^18^F]FEPPA in the brain. The percentage of plasma metabolite is part of the arterial input function for pharmacokinetics modelization.

### 2.5. [^18^F]FEPPA Brain Time Activity Curves

**Figure 7:**
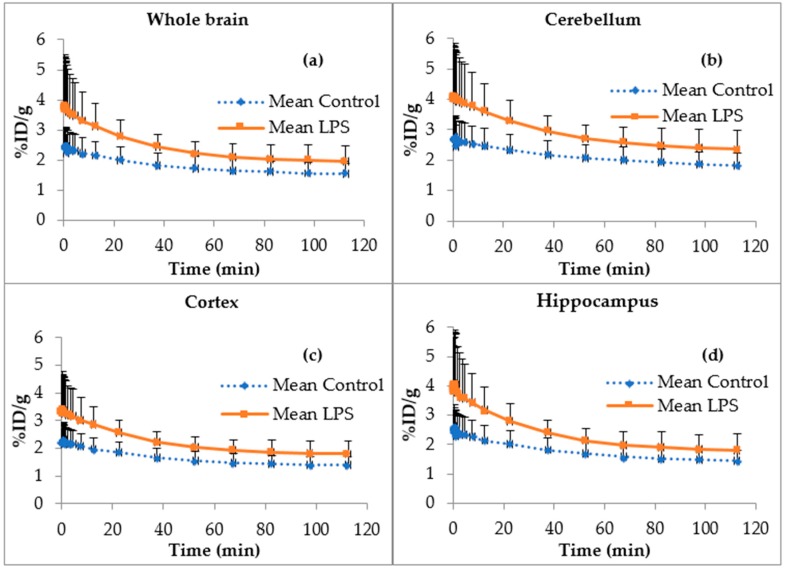
Parent fraction of [^18^F]FEPPA Time Activity Curve in %ID/g ± SD control vs. LPS. (**a**) Mean in whole brain (*p* = 0.0063); (**b**) Mean in cerebellum (*p* = 0.0179); (**c**) Mean in cortex (*p* = 0.0126); (**d**) Mean in hippocampus (*p* = 0.0266) (*n* = 8 controls and 7 LPS).

The regional [^18^F]FEPPA time activity curves corrected for radiometabolites for whole brain, cerebellum, cortex and hippocampus (Figure 7) showed a significantly higher cerebral distribution of [^18^F]FEPPA in LPS mice as compared to controls (area under curve (AUC) in different regions, LPS vs. control, *t*-test two-sided, *p* = 0.0063, *p* = 0.0179, *p* = 0.0126 and *p* = 0.0266, respectively). This significant increase of [^18^F]FEPPA AUC is consistent with the overexpression of its target in brain of LPS-injected mice despite the known and observed variability of the biological effect of LPS. The lack of reference tissue requires a pharmacokinetic modelling to compare [^18^F]FEPPA brain distribution.

In accordance with data of the literature [41,42,43], we used a two tissue compartment plus vascular trapping model in this study. In this model, *K*_1_ and *k*_2_ represent passage (through the blood-brain barrier) from the blood to a nonspecific compartment (free + non specific fixation), *k*_3_ and *k*_4_ are enter and exit from the non specific compartment to the specific one. *K*_b_ represents slow binding of the radiotracer to the endothelium of vasculature. The most relevant parameter to assert a modification in the distribution is the distribution volume *V_T_*. In our study, the pharmacokinetics parameters in the whole mice brain (Table 1) showed a significant increase for *K*_1_, *V_T_* and AUC for LPS mice as compared to control mice (*t* test ± Welch’s correction, one-sided, mice with a %SE > 50% for at least one parameter were excluded). An increase in *k*_3_/*k*_4_ was also observed, although not significant.

## 3. Discussion

In this study, we developed and optimized an easy-to-perform automated [^18^F]FEPPA radiosynthesis process suitable for clinical use based on previous work of Vasdev et al. [33]. Our study also documented plasma and brain metabolism of [^18^F]FEPPA in mice, allowing for demonstration of significant changes in the brain distribution of unmetabolized [^18^F]FEPPA in a murine model of peripheral inflammation using ip LPS injection. 

In this radiosynthesis, a less toxic solvent was used in the mobile phase for semi-preparative HPLC than previous studies. Indeed, according to ICH (Q3C) and the FDA, ethanol is a class 3 solvent, whereas methanol, used previously [31,33], is class 2 and should be limited in pharmaceuticals due to its inherent toxicity. Use of methanol requires an additional reformulation step such as SPE cartridge capture [33] or drying and recovery with ethanol/water [31]. Besides, it still leaves residual traces of solvent in the final product and so requires residual solvent quantification, which is time-consuming and costly. Our new radiosynthesis conditions achieved a slightly better non-decay corrected radiochemical yield of 39% ± 3% in 34 min, as compared to 30% ± 6% in 36 min reached by Vasdev et al. [33], from end of azeotropic drying to the end of formulation. Berroterán-Infante et al. [34] decribed an optimized radiosynthesis process with a high nondecay corrected radiochemical yield of 38% ± 3% (EOB) but using acetonitrile for purification and followed by formulation with cartridge. Another study has reported a radiosynthesis process using also ethanol and water in the purification and formulation processes with a lower radiochemical yield of 13 ± 8% in 59 min (EOB) [35]. Our optimized radiosynthesis process use ethanol, water and phosphoric acid for purification allowing us to bypass the formulation step. This method achieved a nondecay corrected radiochemical yield of 34% ± 2% (EOB) in 55 min.

[^18^F]FEPPA was produced with high radiochemical, chemical purity, and molar activity comparable to those reported in the literature [33,34] with class 3 solvent (ethanol/water) for purification. 

To evaluate this radiotracer, a dynamic µPET/CT imaging study was carried out in vivo with a mouse model of inflammation induced by ip injection of 5 mg/kg of LPS as compared to saline-injected control mice. The intrastriatal injection of LPS or AMPA in mice or rats is a known model of brain inflammation. For example, a study using three differents PET TSPO radiotracers ([^18^F]DPA-714, [^11^C]PK11195, and [^18^F]GE-180) in rats that have been stereotactically injected with 1 μg LPS in the right striatum have shown that [^18^F]GE-180 was able to shows a higher core/contralateral ratio and BP_ND_ when compared to (R)-[^11^C]PK11195, while [^18^F]DPA-714 did not [44]. However, the act of introducing a needle into the brain itself may cause local inflammation that could interfere with the experiment. We therefore chose a less invasive and easier model consisting of a single ip injection of LPS (5 mg/kg). The *Salmonella enterica* serovar *typhimurium* LPS inflammation model is less documented than *E. coli* LPS model, and some studies suggest differences in the inflammation pathways induced. Indeed, *Salmonella enterica* serovar *typhimurium* LPS induces a greater increase in inflammatory cytokines through toll-like receptor TLR2 [45,46]. Western blot analysis showed a significant 2.2-fold increase in brain TSPO expression in the whole brain of LPS-injected mice as compared to control mice 24 h after LPS injection (Figure 6) which allowed to validate a positive control for the TSPO-PET brain imaging study. 

Alongside dynamic PET/CT imaging with [^18^F]FEPPA, the metabolism study showed the relatively important and rapid metabolism of the radiotracer in plasma, in accordance with previous studies [33]. Indeed, our metabolism study documented one main metabolite and a rapid decrease of the parent fraction in plasma (Figure 6). In contrast, our study showed that [^18^F]FEPPA brain metabolism was less important than in the plasma, which is in accordance with one previous study in rats [31]. This metabolism in the plasma may be a disadvantage in terms of [^18^F]FEPPA use since it makes its quantification more difficult. However, most of the TSPO radioligands available are also known to shown significant metabolism. For example, [^18^F]DPA-714 parent fraction represents 11–15% of total radioactivity in the blood 120 min after injection in rats and baboons [47]. 

[^18^F]FEPPA biodistribution in C57BL6 mice confirmed previously published results in rats and athymic mice: rapid uptake by the lungs, heart, and spleen and excretion by the kidneys [31,33]. The whole body distribution of [^18^F]FEPPA (Figure 4) show the relative low uptake in brain as compared to peripheral tissue like heart or kidney as it was expected for a TSPO radiotracer [39]. The low [^18^F]FEPPA brain uptake in our control mice (%ID/g in brain within 2–4%) is rather favorable for signal/background ratio i.e., the detection of cerebral processes (Figure 4). Furthermore, our study suggests that the time activity curve (TAC) of [^18^F]FEPPA corrected from brain metabolism is significantly increased in the LPS-injected mouse group as compared to control mice (Figure 7). This result could be explained on one hand by the significant increase of the specific target TSPO protein expression in the brain of LPS-injected C57Bl6 mice as compared to control group, as confirmed by western blot analysis, and on the other hand by an opening of the blood-brain barrier (BBB) [38]. BBB disruption was shown in some brain regions sush as frontal cortex, thalamus, or cerebellum in male CD1 mice (6–10 weeks old) 24 h after one single ip injection of LPS 3 mg/kg [38].

This significant increase in [^18^F]FEPPA distribution in the brain of LPS-injected mice as compared to control mice was observed consistently throughout the main brain structures/regions such as whole brain, cortex, cerebellum, and hippocampus (Figure 7). 

Pharmacokinetic parameters for [^18^F]FEPPA distribution in the whole brain of mice showed a significant increase in the total distribution volume *V_T_* in LPS-injected mice as compared to control mice. There were no other statistically significant changes in constant rates between the plasma, brain and vascular compartments (Table 1), except for the *K*_1_, which was significantly increased in the LPS group. *K*_1_ is the uptake rate constant for compartment 1 which represents free and non-specifically bound tracer. This increase in *K*_1_ suggests improved [^18^F]FEPPA passage across the blood-brain barrier in LPS-injected mice which could be explained by a BBB disruption. *K_b_* (the rate of binding of the radiotracer to the endothelium of the vasculature [41,42]) was not modified between LPS and control mice. This therefore suggests that the increased [^18^F]FEPPA *V_T_* observed in the brain is more likely due to an increase in the TSPO expression in the brain parenchyma of LPS-treated mice. The *k*_3_/*k*_4_ ratio, also named BP_ND_, is the ratio at equilibrium of specifically-bound radioligand to non-displaceable radioligand in the tissue. The increased *k*_3_/*k*_4_ ratio in the LPS-treated mice suggests higher specific binding to TSPO. The fact that this increase in the *k*_3_/*k*_4_ ratio is not statistically significant is possibly due to its high variability. This high *k*_3_/*k*_4_ ratio variability we have observed is consistent with the relatively high standard deviation observed in [^18^F]FEPPA TAC especially in the brain of LPS injected mice group (Figure 7). Indeed Rusjan et al. have reported that *k*_3_/*k*_4_ ratio is highly variable, and the most reliable parameter to document an increase in [^18^F]FEPPA brain distribution is the increase in *V_T_* [48]. Furthermore, the same team has reported, by Monte-Carlo simulation, that an increase or decrease of *K*_1_ does not significantly modify *V_T_* [48]. 

The hypothesis in a drug/compound brain distribution increase linked to a higher cerebral blood flow could be true only for molecules that are highly extracted by the brain. Indeed, according to the Renkin and Crone equation [49], one of the main pharmacokinetic principles of the distribution of molecules in tissues is that it is governed by blood flow and the ratio of blood concentration to tissue concentration. Depending on the value of this ratio, molecules behave in two ways: (i) for molecules that are poorly extracted by brain tissue (<30%), variations in blood flow have little or no influence on their brain distribution; these molecules are called blood-flow independent or permeability dependent; (ii) for molecules that are strongly extracted by brain tissue (>30%), such as glucose or oxygen, cerebral blood flow strongly influences their brain distribution. [^18^F]FEPPA is a molecule with low brain extraction (2 to 4% ID/g), and the cerebral passage of this type of molecule does not depend on the cerebral blood flow. Similarly, the brain distribution of [^11^C]verapamil, which also has low brain uptake between 1.5 and 3% of the injected dose, did not depend on cerebral blood flow [50]. Besides, in a clinical study in healthy subjects, Rusjan et al. have shown that *V_T_* is the most robust parameter for quantifying [^18^F]FEPPA uptake and that it is not modified by an increase or decrease in cerebral blood flow [48].

The increase in [^18^F]FEPPA *k*_3_/*k*_4_ (although not significant) and *V_T_* in the brains of LPS-treated mice is in accordance with an activation of pro-inflammatory microglial state with TSPO overexpression as confirmed by western-blot analysis. This phenotype overexpresses TSPO while reparative and mixed stages do not [51]. [^18^F]FEPPA is one of the main second generation TSPO radioligands being the most advanced in clinical development to study the neuroinflammation process. It represents a useful tool to follow neuroinflammation in longitudinal study. The [^18^F]FEPPA radio-synthesis method seems easier than that of [^18^F]DPA-714, and the metabolism of [^18^F]FEPPA is less important than that of [^18^F]DPA-714 [47], which is more favorable for [^18^F]FEPPA quantification in brain. However the dynamic acquisition and the three patterns of binding affinity in human are hindrance (both for [^18^F]DPA-714 and [^18^F]FEPPA) to their clinical use.

To summarize, in this study, we have developed an automated synthesis method for [^18^F]FEPPA that includes HPLC purification/formulation easily performed on the Allinone^®^ radiosynthetiser with a nondecay corrected radiochemical yield of 34% ± 2% (EOB) in 55 min and purification with only class 3 solvent (ethanol). The [^18^F]FEPPA radiotracer obtained is more suitable for preclinical and clinical use than those obtained before. The PET imaging study using the TSPO ligand [^18^F]FEPPA has allowed us to observe a significant increase in [^18^F]FEPPA *V_T_* in the brain of a murine neuroinflammation model 24 h after a single ip injection of *Salmonella enterica serovar typhimurium* LPS.

## 4. Materials and Methods 

### 4.1. Radiochemical Synthesis of [^18^F]FEPPA

#### 4.1.1. General

All reagents and solvents were purchased from commercial suppliers (ABX, Radeberg, Germany or Sigma-Aldrich, St Quentin Fallavier, France) and were used without further purification. Sep-Pak QMA were purchased from ABX. [^18^F]fluoride ion was produced via the [^18^O(p,n)^18^F] nuclear reaction (IBA Cyclone 18/9 cyclotron).

Radioactivity of the final product was measured with a dose calibrator (PET DOSE 5 Ci, COMECER^®^, Castel Bolognese, Italy).

#### 4.1.2. Radiosynthesis

**Figure 8:**
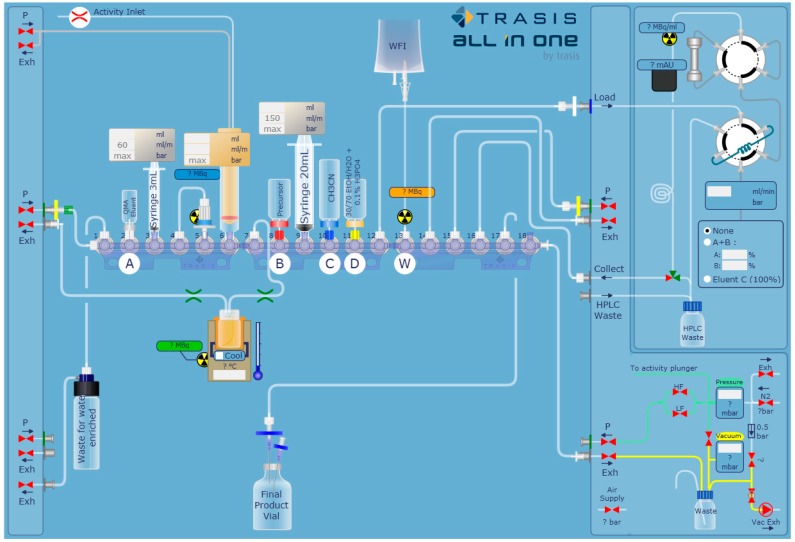
[^18^F]FEPPA radiosynthesis layout on an AllInOne^®^ module.

Radiosynthesis of [^18^F]FEPPA was performed using an AllInOne^®^ (Trasis, Ans, Belgium) synthesis module and a tosylated precursor for a one-step fluorine nucleophilic aliphatic substitution, based on the radiosynthesis previously described [31] (Figure 8).

The list of reagents used in the automated procedure is presented in Table 2. [^18^F]FEPPA was synthesized using an in-house reaction sequence described in Table 3.

The aqueous [^18^F]fluoride target solution was loaded on a QMA (Pre-conditioned Sep-Pak^®^ Light QMA cartridge, ABX). The concentrated [^18^F]fluoride was eluted into the reactor using a K_2_CO_3_ (3 mg) and Kryptofix (K_222_, 15 mg) mixed solution (1 mL, CH_3_CN/H_2_O, 80/20, *v*/*v*). The solvents were evaporated under reduced pressure at 110 °C for 10 min. To the dry residue containing the K222/potassium [^18^F]fluoride complex was added the tosylated precursor (2-(2-((N-4-phenoxypyridin-3-yl)acetamido)methyl)phenoxy)ethyl 4-methylbenzenesulfonate) (5 mg) in acetonitrile (1 mL), and the mixture was heated and maintained at 90 °C for 10 min. After cooling, the radiolabeling reaction is then stopped by the addition of 3 mL of mobile phase 30/70 *v*/*v* ethanol/water + 0.1% of phosphoric acid in the reactor. After passing through a 0.22 μm vent filter, this solution is transferred into the injection loop and followed by rinsing with 3 mL of mobile phase. The purification by semi-preparative HPLC (column Phenomenex Kinetex^®^ C18 10 μm 250 × 10 mm; Le Pecq, France) is carried out in isocratic with a solution (30/70 *v*/*v*) of ethanol/water + 0.1% of phosphoric acid at 4 mL/min. The fraction containing [^18^F]FEPPA, associated to a well-defined radioactive peak, was collected at 13–14 min. The finished product is then received in a flask containing 1.5 mL of sodium bicarbonate 8.4%. The radiotracer solution was finally passed through a 0.22 μm Millipore filter into a sterile vial for in vivo experiments. Production was diluted in saline to reach a volume activity of 1.8–2 GBq/mL.

#### 4.1.3. Quality Control

The radiochemical and chemical purity, stability, and molar activity measurements were performed by analytical HPLC. The molar activity of the radiotracer was assessed by measurement of the injected radioactivity, and the FEPPA concentration in the sample was derived from the UV detection.

The identity of the labeled compound [^18^F]FEPPA was confirmed by co-injection with a non-radioactive standard of FEPPA. The FEPPA concentration in the radioactive sample was obtained using the UV-peak area ratio between the radioactive product and the standard solution. 

Analytical HPLCs were performed on a Dionex Ultimate 3000 (Thermofisher Scientific, Waltham, MA, USA) with a multi-wavelength UV Diode Array Detector (DAD) in series with a Bioscan gamma detector (Canberra, St Quentin Yvelines, France) using a Kinetex^®^ C18 column (50 × 2.1 mm, 2.6 μm) H_2_O/CH_3_CN 75/25 at 0.6 mL/min. A delay time of 20 s was observed between the two detectors.

### 4.2. Animal Models

All animal experiments were performed in accordance with European guidelines for care of laboratory animals (2010/63/EU) and were approved by the Animal Ethics Committee of Paris Nord.

In vivo quality control of [^18^F]FEPPA (biodistribution studies) was performed in 12-week-old C57Bl/6JRj mice (Janvier, France) (*n* = 16). Eight mice were injected with 500 µL 0.9% NaCl by ip, and 8 with LPS from *salmonella enterica* serotype typhimurium (Sigma-Aldrich, Saint Louis, MO, USA) 5 mg/kg in 500 µL 0.9% NaCl. Micro PET/CT imaging was held 24 h after the LPS injection. Brains were removed immediately after imaging and preserved for western blot. Metabolism study was performed in 12-week-old C57Bl/6JRj mice (Janvier, France) (*n* = 16).

### 4.3. Western Blot Analysis of TSPO

Adult mice brains were dissected and reduced in powder at −80 °C, immediately dissolved in PBS with 2% SDS, and 1× EDTA-free Complete Protease Inhibitor (Roche, Basel, Switzerland). Lysates were sonicated twice at 10 Hz (Vibra cell VCX130) and centrifuged for 30 min at 16,000 rcf at 4 °C. Supernatants were boiled in 5× Laemmli loading buffer. Protein content was measured using the BCA protein assay reagent (Thermo scientific, Waltham, MA, USA). Equal amounts of proteins (20 µg) were separated by denaturing electrophoresis in NuPAGE 4–12% Bis-Tris acetate gradient gel (Invitrogen, Carlsbad, CA, USA) and electrotransfered to nitrocellulose membranes. Membranes were analyzed using the following primary antibodies: rabbit anti-TSPO (Abcam, Cambridge, UK) (1:10,000); horseradish-peroxidase-conjugated (HRP) anti-GAPDH (1:10,000); Secondary antibody used was HRP-conjugated anti-rabbit antibodies (Amersham) (dilution 1:2000). HRP activity was visualized by enhanced chemiluminescence (ECL) using Western Lightning plus enhanced chemoluminescence system (Perkin Elmer, Waltham, MA, USA). Chemoluminescence imaging was performed on a LAS4000 (Fujifilm, Tokyo, Japan). GAPDH expression was used as a loading reference.

### 4.4. Metabolism Study

Adult mice were injected with 68.6 MBq ± 4.4 MBq of [^18^F]FEPPA in the tail vein. Arterial blood sampling (intra cardiac) was done at different times (5, 15, 30, 45, 60, 75, 90, and 120 min) then followed by exsanguination and brain removal under anesthesia (ketamine/xylazine lethal dose). Samples were homogenized with CH_3_CN and then centrifuged. Pellet and supernatant were counted separately on a gamma counter (WIZARD2^®^, Perkin-Elmer, Villebon-sur-Yvette, France).

Radiometabolites in supernatant were performed in HPLC on a Dionex Ultimate 3000 (Thermofisher Scientific, Courtaboeuf, France) with a multi-wavelength UV Diode Array Detector (DAD) in series with a Bioscan gamma detector (Canberra, St Quentin Yvelines, France) with a Kinetex^®^ column (C18 250 × 4.6 mm, 5 µm), gradient H_2_O/CH_3_CN 60/40 to 50/50 in 5 min at 1 mL/min.

### 4.5. In Vivo PET/CT Imaging

PET/CT imaging was performed using Inveon micro PET/CT scanner (Siemens Medical Solutions, Erlangen, Germany) designed for small laboratory animals. Mice were anesthetized (isoflurane/oxygen, 2.5% for induction at 0.8–1.5 L/min, and 1–1.5% at 0.4–0.8 L/min thereafter) during injection of [^18^F]FEPPA (9.9 ± 1.5 MBq) in a volume of 0.15 mL (0.22 ± 0.19 nmol of FEPPA) via the tail vein, and during PET/CT acquisitions.

For [^18^F]FEPPA biodistribution studies, dynamic mod-list PET acquisitions were performed from time of radiotracer injection until 120 min after injection (*n* = 16). The dynamic list-mode contains 25 frames (5 s × 12; 60 s × 4; 5 min × 2; 15 min × 7). 

The spatial resolution of Inveon PET device was 1.4 mm full-width at half-maximum at the center of the field of view. Images were reconstructed using a 3D ordered subset expectation maximization method including corrections for scanner dead time, scatter radiations, and randoms.

### 4.6. Data Analysis and Modeling

PET/CT images were visually assessed. Then quantitative analysis of PET/CT images was performed by PMOD version 3.806 image analysis software (PMOD Technologies, Zurich, Switzerland). For comparisons, all values of radioactivity concentrations were normalized by the injected dose and expressed as percentage of the injected dose per g of tissue (% ID/g).

PET images were automatically rigid matched with corresponding CT and then cropped to keep only the brain images. CT were automatically rigid matched with a T2 MRI template (M. Mirrione, included in PMOD), and then the transformation applied to the PET image was cropped. This method allowed us to use the atlas of the brain corresponding to the T2 MRI template.

The arterial input function was computed from plasma sampling and corrected for metabolism of the parent ligand. A two compartment + vascular trapping 4 rate-constant kinetic model was used to characterize [^18^F]FEPPA pharmacokinetics as previously described [41,42]. Model parameters were estimated for influx constant *K*_1_ (mL·cm^−3^·min^−1^), efflux (*k*_2_) (min^−1^) rate of radioligand diffusion between plasma and brain compartment. Exchange between compartments *k*_3_ and *k*_4_ (min^−1^) were also estimated. Finally, the *K_b_* (min^−1^) parameter that describes the rate of binding to the TSPO in the endothelium and the macro-parameters *V_T_* (mL·cm^−^^3^) was also used to estimate the total distribution volume for the whole brain with goodness of fit evaluated by inspection [43]. 

### 4.7. Statistical Analysis

Data are presented as mean ± SD. Statistical analysis was performed using Prism 5, version 5.0.1 (La Jolla, CA, USA). A significance value of *p* < 0.05 was used. 

## Figures and Tables

**Table 1:** Pharmacokinetics parameters for [^18^F]FEPPA in the whole mice brain.

Group	*K*_1_ (mL·cm^−3^·min^−1^)	*k*_2_ (min^−1^)	*k*_3_/*k*_4_	*K*_b_ (min^−1^)	*V_T_* (mL·cm^−3^)	AUC _0 to 120 min_ (%ID/g s^−1^)
Control (*n* = 6)	0.58 ± 0.15	0.35 ± 0.06	0.34 ± 0.13	0.53 ± 0.05	2.25 ± 0.44	11,910 ± 934
LPS (*n* = 5)	0.86 ± 0.18	0.36 ± 0.13	0.61 ± 0.58	0.68 ± 0.23	3.77 ± 0.41	15,940 ± 1226
*p*	0.0112	0.3210	0.1818	0.1092	0.0001 ***	0.0032 **

** *p* < 0.001, *** *p* < 0.0001.

**Table 2:** List of reagents.

Position	Reagents	Quantities
5	Pre-conditioned Sep-Pak^®^ Light QMA	1
2 (vial A)	Eluent QMA (K_2_CO_3_/K_222_ in CH_3_CN/H_2_O, 80/20, *v*/*v*)	1 mL
8 (vial B)	Precursor	5 mg
10 (vial C)	CH_3_CN anhydrous	15 mL
11 (vial D)	Mobile phase (30/70 EtOH/H_2_O + 0.1% phosphoric acid)	6 mL
13 (bag W)	WFI	250 mL

**Table 3:** Reaction sequence for [^18^F]FEPPA radiosynthesis.

**Fluorination of Precursor**
1. [^18^F]fluoride trapping on a pre-activated QMA cartridge 2. [^18^F]fluoride desorption by eluent 3. Azeotropic evaporation at 110 °C for 10 min 4. Addition of precursor to the reactor vial 5. [^18^F]fluorination at 90 °C for 10 min 6. Cooling the reactor vial 7. Addition of HPLC mobile phase to the reactor vial
**Purification of [^18^F]FEPPA**
1. Injection on HPLC semi-preparative 2. Collection of [^18^F]FEPPA in 1.5 mL 8.4% sodium bicarbonate
**Formulation of [^18^F]FEPPA**
1. Dilution of the collected fraction with NaCl 0.9% 2. Sterile filtration
